# REpeated AutoLogous Infusions of STem cells In Cirrhosis (REALISTIC): a multicentre, phase II, open-label, randomised controlled trial of repeated autologous infusions of granulocyte colony-stimulating factor (GCSF) mobilised CD133+ bone marrow stem cells in patients with cirrhosis. A study protocol for a randomised controlled trial

**DOI:** 10.1136/bmjopen-2015-007700

**Published:** 2015-03-20

**Authors:** A King, D Barton, H A Beard, N Than, J Moore, C Corbett, J Thomas, K Guo, I Guha, D Hollyman, D Stocken, C Yap, R Fox, S J Forbes, P N Newsome

**Affiliations:** 1 NIHR Centre for Liver Research and Biomedical Research Unit, University of Birmingham, Birmingham, UK; 2 Liver Unit, University Hospital Birmingham NHS Foundation Trust, Birmingham, UK; 3 NIHR Liver BRU Clinical trials group (EDD), CRUK clinical trials unit, University of Birmingham, Birmingham, UK; 4 Cellular and Molecular Therapies, NHS Blood and Transplant, Birmingham, UK; 5 MRC Centre for Regenerative Medicine, University of Edinburgh, Edinburgh, UK; 6 National Institute for Health Research Biomedical Research Unit in Gastrointestinal and Liver Diseases at Nottingham University Hospitals NHS Trust and the University of Nottingham, Nottingham, UK; 7 Newcastle Clinical Trial Unit, Institute of Health and Society, Newcastle University, Newcastle, UK

## Abstract

**Introduction:**

Liver disease mortality and morbidity are rapidly rising and liver transplantation is limited by organ availability. Small scale human studies have shown that stem cell therapy is safe and feasible and has suggested clinical benefit. No published studies have yet examined the effect of stem cell therapy in a randomised controlled trial and evaluated the effect of repeated therapy.

**Methods and analysis:**

Patients with liver cirrhosis will be randomised to one of three trial groups: group 1: Control group, Standard conservative management; group 2 treatment: granulocyte colony-stimulating factor (G-CSF; lenograstim) 15 µg/kg body weight daily on days 1–5; group 3 treatment: G-CSF 15 µg/kg body weight daily on days 1–5 followed by leukapheresis, isolation and aliquoting of CD133+ cells. Patients will receive an infusion of freshly isolated CD133+ cells immediately and frozen doses at days 30 and 60 via peripheral vein (0.2×10^6^ cells/kg for each of the three doses). Primary objective is to demonstrate an improvement in the severity of liver disease over 3 months using either G-CSF alone or G-CSF followed by repeated infusions of haematopoietic stem cells compared with standard conservative management. The trial is powered to answer two hypotheses of each treatment compared to control but not powered to detect smaller expected differences between the two treatment groups. As such, the overall α=0.05 for the trial is split equally between the two hypotheses. Conventionally, to detect a relevant standardised effect size of 0.8 point reduction in Model for End-stage Liver Disease score using two-sided α=0.05(overall α=0.1 split equally between the two hypotheses) and 80% power requires 27 participants to be randomised per group (81 participants in total).

**Ethics and dissemination:**

The trial is registered at Current Controlled Trials on 18 November 2009 (ISRCTN number 91288089, EuDRACT number 2009-010335-41). The findings of this trial will be disseminated to patients and through peer-reviewed publications and international presentations.

**Strengths and limitations of this study**

- Large randomised controlled trial of haematopoietic stem cells in patients with liver cirrhosis.
- Effects of granulocyte colony-stimulating factor are tested in a separate arm from cell infusions.
- Patients study are well-defined (cirrhosis) within narrow Model for End-stage Liver Disease scores (11.50–15.49).
- Randomisation is stratified by disease aetiology and recruitment site.
- Mixture of aetiologies of liver disease.
- No information on amount of haematopoietic stem cells homing to the injured liver.

## Introduction

Chronic liver disease is the fifth commonest cause of death in the UK whose incidence is rising predominantly due to alcohol consumption, obesity and hepatitis C virus infection. At present liver transplantation is the only curative treatment for end-stage liver disease however this is limited by donor organ availability, the supply of which has not matched the rising number of patients requiring liver transplantation.^1^ ^2^ Moreover, liver transplantation requires long-term immunosuppression which is associated with increased risk of cardiovascular disease, renal impairment and malignancy.^3^ There is a clear need for new therapies for chronic liver disease which are either an effective alternative to liver transplantation or reduce the proportion of patients that require transplantation.

Initial observations that the bone marrow may contribute to hepatic repair and regeneration^4–6^ were followed by studies of the therapeutic benefits of bone marrow derived cells in animal models of liver injury. Bone marrow (BM) cells injected into mice with chronic liver injury resulted in a reduction in hepatic fibrosis and an improvement in serum albumin,^7^ while mobilisation of BM cells by injection of GCSF reduced the severity of liver injury and improved survival in both acute and chronic models of liver injury.^8^ However, other studies have shown either no beneficial effect of BM cell injections^9^ or even a worsening of hepatic fibrosis after bone marrow cell injection.^10^

Despite varied outcomes in preclinical studies, multiple clinical studies of BM-derived stem cell therapy have been performed. Am Esch and colleagues studied patients without chronic liver disease undergoing liver resection for metastatic cancer affecting the liver. They demonstrated accelerated hepatic regeneration in patients receiving infusions of BM-derived CD133+ stem cells with concomitant portal vein embolisation prior to hepatic resection.^11–13^ In patients with liver disease trials of bone marrow stem cell therapy have included small numbers of patients and have been designed to examine safety and feasibility rather than efficacy.^3^ ^14^

The initial reports of the use of BM-derived stem cells in patients with chronic liver disease utilised a variety of approaches to the method of cell isolation and route of delivery. Gordon *et al*^15^ administered GCSF to patients with cirrhosis and collected CD34+ haematopoietic stem cells (HSC) mobilised into the blood and infused them directly into the liver via the hepatic artery. Alternatively, investigators in another study directly harvested BM cells from the iliac crest under general anaesthesia, selected out mononuclear BM cells and infused these cells via the portal vein.^16^ While both studies involved only small numbers of patients, 5 and 9, respectively and lacked control participants, they demonstrated that this form of therapy appeared safe and feasible. Clinical outcomes were reported in both studies and showed reduced serum bilirubin and improved serum albumin 2 months following cell infusion in the first study^15^ and improved serum albumin and Child-Pugh score (a marker of liver dysfunction) at 6 months in the second.^16^ These two initial studies highlight the significant number of variables involved in comparing these and subsequent studies.

Further small-scale, uncontrolled studies have included variations in the patients enrolled (aetiology and severity of liver disease), the method of cell harvest (direct BM aspiration or peripheral blood leukopharesis), the type and number of cells infused (various populations from unsorted mononuclear cells to purified CD133+ HSC) and the route of cell infusion (hepatic artery, portal vein or peripheral vein).^3^ The aim of many of these studies was to determine safety and feasibility and in that sense the outcomes were promising. However, the size and nature of many of these subsequent studies meant that meaningful conclusions on clinical outcomes could not be drawn.

In more recent small controlled trials, Lyra *et al*^17^ demonstrated significant improvements in liver function 1 year following infusion of BM mononuclear cells into the hepatic artery when compared with untreated control patients.^18^ Ismail *et al*^19^ examined the role of infusion of bone marrow mononuclear cells prior to liver resection in patients with cirrhosis with hepatocellular carcinoma and showed improved outcomes 3 months postoperatively compared with patients undergoing resection alone.

There have been many reports of clinical studies describing the use of BM stem cells and HSC in patients with chronic liver disease, which have predominantly involved small numbers of patients and made no comparisons with untreated controls. These studies were systematically reviewed by Moore *et al*^20^ and of the studies considered only one^21^ was found to be of adequate trial design to be informative regarding the potential efficacy of cell therapy and this trial was negative. The general message from these studies is that stem cell therapy appears to be a safe and feasible therapeutic option, however its efficacy has yet to be proven. There is a need for larger, randomised, controlled studies of stem cell therapy for liver disease with powered primary end points.

## Research aim

The REALISTIC trial will evaluate the efficacy and safety of CD133+ BM-derived HSC in patients with compensated cirrhosis. The aim of the study is to demonstrate an improvement in liver function and a reduction in liver fibrosis in patients receiving stem cell therapy compared with those receiving standard management of compensated cirrhosis.

## Methods and analysis

The REALISTIC trial is a multicentre, early phase, open-label randomised controlled trial of two different therapies—(1) administration of G-CSF alone and (2) administration of G-CSF, isolation of CD133+ BM HSC followed by repeated infusion of CD133+ cells, compared with standard management of compensated cirrhosis according to local, national and international guidelines.

## Trial organisation

The REALISTIC trial is an investigator led and designed trial, co-ordinated by the Liver Research Group within the Cancer Research Clinical Trials Unit at the University of Birmingham. The University of Birmingham is the trial sponsor and the trial is funded by the NIHR Biomedical Research Unit for Liver Disease, Birmingham and the Sir Jules Thorn Trust. The trial is run at three sites in the UK (Birmingham, Edinburgh and Nottingham) and recruitment began in May 2010.

The trial was registered at Current Controlled Trials (http://www.controlled-trials.com) on 18 November 2009 (ISRCTN number 91288089, EuDRACT number 2009-010335-41).

The procurement, processing, storage and distribution of the Autologous CD133+ HSC is performed in accordance with Tissue Quality and Safety Regulations by establishments holding Human Tissue Authority licences.

## Inclusion and exclusion criteria

Patients eligible for the trial are those aged 18–70 with compensated cirrhosis and a Model for End-stage Liver Disease (MELD) score greater than or equal to 11.5 and less than 15.5. The specific inclusion criteria are listed in table 1.

**Table 1:** Inclusion criteria

Criteria	Details
Age	18–70
Cirrhosis	Previous liver biopsy confirming histological features of cirrhosis
	Transient elastography (Fibroscan) >18 kPa
	Clinical and radiological features that in the opinion of the investigator are in keeping with a diagnosis of cirrhosis
	AST:Platelet Ratio Index (APRI) >2
Aetiology of Liver Disease	Alcohol-related liver disease: Features (clinical, biochemical, histological or radiological) of chronic liver disease with a compatible history of alcohol excess (>80 g/day), in the absence of other causes of chronic liver disease Abstinent for ≥6 months prior to enrolment
	Chronic hepatitis C virus infection, positive HCV Antibody +/− PCR positive for HCV RNA Not currently on antiviral therapy
	Chronic hepatitis B virus infection Positive HBsAg and Anti-HBC Established on antiviral therapy with adequate viral suppression
	Primary biliary cirrhosis Two out of: cholestatic LFTs, positive AMA (>1:40),compatible histology If already receiving ursodeoxycholic acid: must be established on current dose >3 months prior to enrolment
	Genetic haemochromatosis Diagnosis made on basis of compatible biochemistry (transferrin sat >60%, ferritin >400), genotype (homozygous C282Y or H63D, compound heterozygote) or Histology
	Cryptogenic cirrhosis Diagnosis of cirrhosis unattributable to any other cause
	Non-alcoholic fatty liver disease Either: histological evidence of steatosis in the absence of other liver diseases Or: Imaging compatible with NAFLD (eg Fatty infiltration of liver) and one or more risk factors (eg, elevated BMI, T2DM, hypertriglyceridaemia, hypertension) And: The absence of significant alcohol consumption (<20 g/day) and no evidence of other causes of chronic liver disease
	α-1 anti-trypsin deficiency Diagnosis based on compatible genetic, phenotypic or histological testing
MELD score	11.5–15.5

AMA, antimitochondrial antibody test; BMI, body mass index; HBC, hepatitis C virus; HBsAg, HBV surface antigen; T2DM, type 2 diabetes mellitus; LFTs, liver function tests; MELD, Model for End-stage Liver Disease.

In addition to the general exclusion criteria there are specific exclusion criteria related to liver disease and to the safety of GCSF administration, which are listed in table 2.

**Table 2:** Exclusion criteria

Criteria	Details
General	Refusal or inability to give informed consent
	Any situation that in the Investigator's opinion may interfere with optimal study participation such as alcohol or drug abuse, domicile too distant from study site, potential non-compliance or inability to co-operate
	Participation in any clinical study of an investigational agent within 30 days of randomisation
	The presence of clinically relevant cardiovascular, pulmonary, gastrointestinal, renal, metabolic, haematological, neurological, psychiatric, systemic, ocular, gynaecological or any acute infectious disease or signs of acute illness that in the opinion of the investigator might compromise the patient's safe participation in the study
	Presence or history of cancer within past 5 years with exception of adequately treated localised basal cell carcinoma of the skin, in situ cervical cancer or solid malignancy surgically excised in total without recurrence for 5 years
Pregnancy or Breastfeeding	Women of childbearing potential and men who have partners of childbearing potential who are not willing to practise effective contraception for the duration of the study and for 12 months (females) and 6 months (males) after the last study drug administration
Liver specific	Alcohol ingestion >21 units/week (male) >14 units/week (female)
	Aetiology of chronic liver disease out with those listed in the inclusion criteria
	Ascites—unless minimal and well controlled with no changes to diuretic therapy in the last 3 months
	Encephalopathy—current or requiring hospitalisation in last 3 months
	Portal hypertensive bleeding—active or requiring hospitalisation in past 3 months
	Hepatocellular carcinoma—current or previous
	Liver transplantation—previous or on waiting list
GCSF related	Recent history of pulmonary infiltrates or pneumonia: patients should have completely recovered from any previous episodes, both clinically and radiologically

## Screening and randomisation

Patients will be identified and recruited at the participating trial site and give informed written consent at the beginning of the screening visit prior to undergoing any tests and procedures needed to assess eligibility.

Eligibility for the trial is determined by clinical assessment including a full medical history and clinical examination, baseline blood tests, complete liver screen if not previously completed as part of standard care, 12-lead ECG, abdominal ultrasound sonography and liver stiffness evaluation, baseline chronic liver disease questionnaire (CLDQ) and urinary pregnancy test in women of childbearing age. As required by National Health Service (NHS) Blood and Transplant Standard Operating Procedures, prior to processing and storage of cellular products, patients are tested for hepatitis B virus (HBV), hepatitis C virus (HCV), HIV, human T-cell lymphotropic virus 1 (HTLV1) and (HTLV 2) and syphilis.

Eligible patients will be randomly assigned to one of the three treatment groups on a 1:1:1 basis. To minimise bias randomisation will be stratified by recruiting centre and by aetiology of underlying liver disease (alcohol-related liver disease, chronic HCV and other).

## Study treatment

All patients will receive standard management for a patient with compensated cirrhosis, which may include disease specific medications (eg, ursodeoxycholic acid for primary biliary cirrhosis) and treatments for the complications of cirrhosis (eg β-blockers for prophylaxis of variceal haemorrhage). Concomitant medications are permitted at the discretion of the site investigator with the exceptions of the introduction of antiviral therapy for chronic HCV infection, changes to medications for chronic HBV infection, the introduction of ursodeoxycholic acid for primary biliary cirrhosis and participation in another clinical trial of an investigational product.

Patients randomised to group 2 will begin treatment within 7 days of randomisation and will receive subcutaneous injections of GCSF (lenograstim) 15 µg/kg body weight daily for 5 days, in addition to standard management.

Patients randomised to group 3 will begin treatment within 7 days of randomisation and will receive subcutaneous injections of GSCF (lenograstim) 15 µg/kg body weight daily for 5 days, in addition to standard management. On the fifth day of GCSF treatment leukapheresis will be performed. Leukapheresis will be performed according to the standard operating procedure in place at each trial site and peripheral blood mononuclear cells will be collected. If insufficient peripheral blood mononuclear cells are obtained then a second leukapheresis will then be performed on day 6.

Isolation of CD133+ HSC from the harvested peripheral blood mononuclear cells is performed under aseptic conditions within clean room facilities in accordance with good manufacturing practice regulations (Medicines and Healthcare products Regulatory Agency, MHRA/HTA, UK). CD133+ HSC are isolated through immunomagnetic positive selection using super paramagnetic iron dextran particles directly conjugated to CD133 antibodies. This is performed in a closed, sterile system providing clinical grade enrichment (CliniMACS Plus, Miltenyi Biotec, Germany). The CliniMACS system has been shown to provide CD133+ cells at up to 97% high purity and yields of up to 81%,^22^ ^23^ with passive depletion of unwanted cells. The CliniMACS Plus instrument is CE-marked for clinical use in Europe.

**Figure 1:**
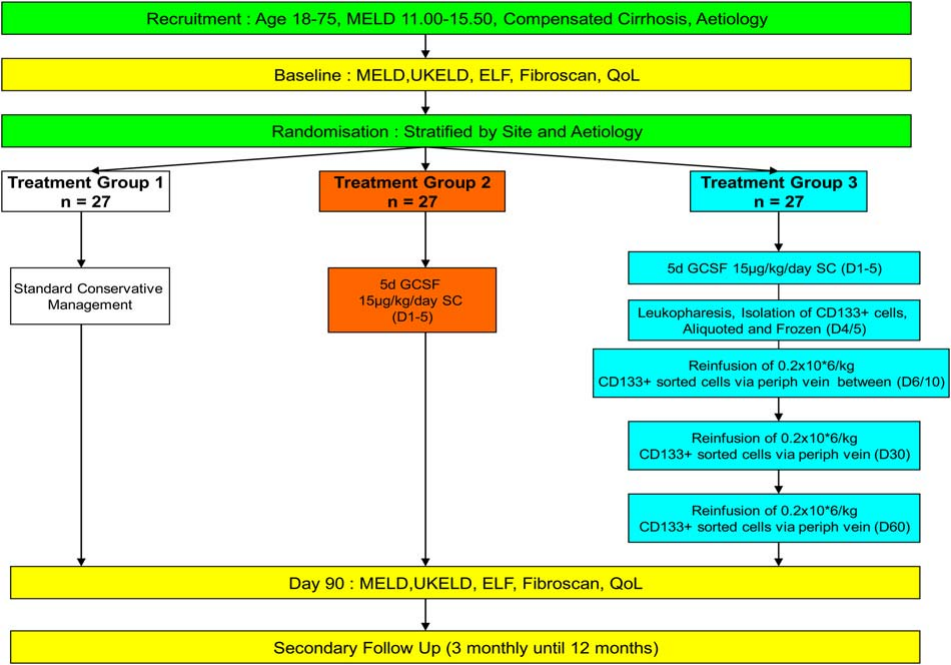
Outline of REALISTIC Trial. Patients are randomised to one of three arms as indicated in the flow chart.

Isolated CD133+ HSC are aliquoted in three portions in the required quantities, one portion will be available for immediate reinfusion and a further two portions will be cryopreserved according to site standard protocols for later reinfusion at day 30 and day 60 postrandomisation (figure 1). The dosage of CD133+ cells to be re-infused is 0.2×10^6^ cells/kg for each of the three infusions at monthly intervals, thus requiring the collection of a minimum of 0.6×10^6^ cells/kg. This dosage is based on maximising the number of cells to be re-infused while ensuring that sufficient cells can be harvested from each patient to standardise the treatment. In the event of there being insufficient cells for three doses then cells will be allocated preferentially to the first, then second and third dose. Levels of 1.2×10^6^ cells/kg were used as a cut-off in order to allow for 50% cell loss during selection.

A recent study examining the effectiveness of peripheral blood stem cell mobilisation and harvesting in patients with cirrhosis demonstrated that while this procedure was safe and effective, the number of cells harvested from cirrhotic patients was lower than in healthy controls. As a mean number of 1.2±0.5×10^6^ cells/kg were collected in the Lorenzini study^24^ the target of 0.6×10^6^ cells/kg in this trial should be achievable. CD133+ HSC thawing and reinfusion is performed according to the site standard protocol for the Reinfusion of Autologous Stem Cells.

## Safety considerations

Prior studies have shown the safety and feasibility of GCSF in the mobilisation of peripheral blood stem cells in patients with cirrhosis and specific safety checks have been included to provide further evaluation. An ultrasound scan to measure the spleen size will be performed on day 5 of GCSF treatment as GCSF administration increases spleen size but the significance of this is not clear in patients with cirrhosis and possible pre-existing splenomegaly. The peripheral blood white cell count will be measured on day 4 of GCSF treatment and treatment discontinued if greater than 70×10^9^ cells/mL. A chest X-ray will be performed prior to administration of GCSF to exclude pre-existing pulmonary infiltrates or other lung diseases.

## Outcome measures

### Primary outcome measure

**Figure 2:**
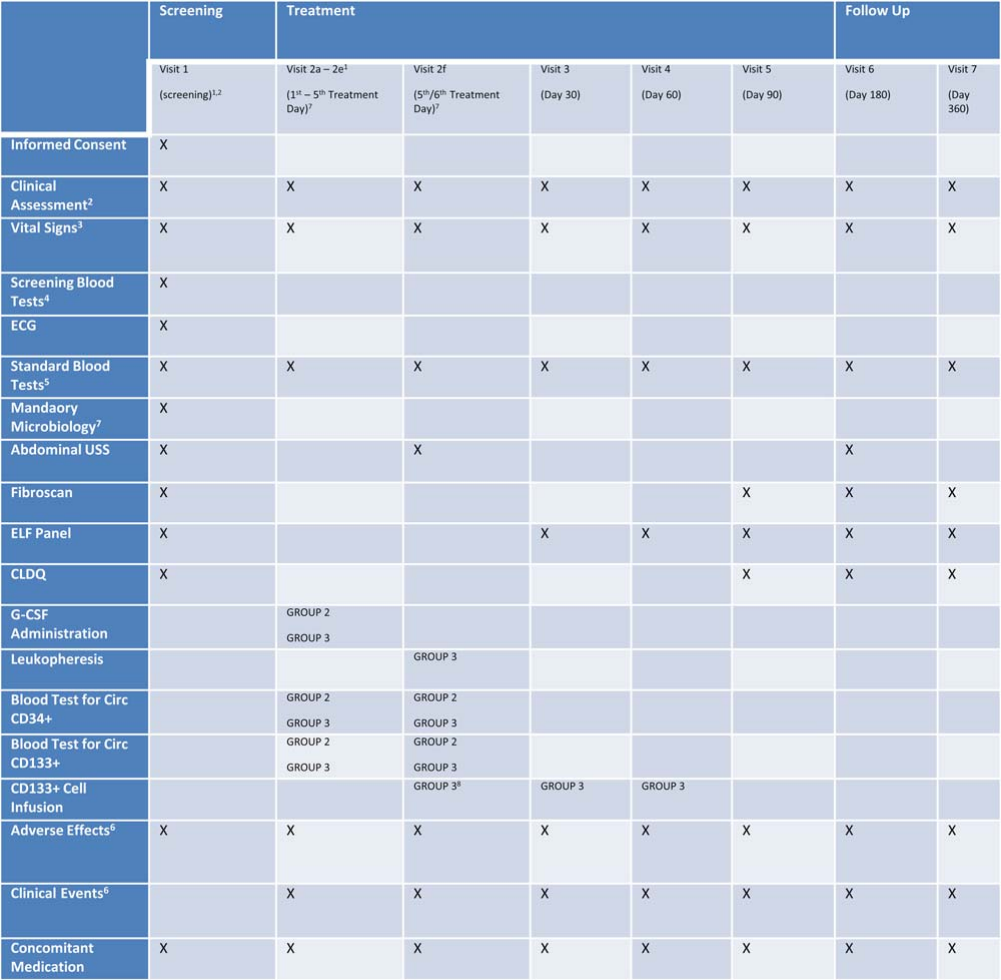
(1) For patients in groups 1 and 2 visit 1 and visit 2a should be combined into 1 day where possible. For patients in group 3, timing of visit 2a will depend on scheduling of leukapheresis. (2) All screening tests must be completed less than 7 days prior to randomisation and treatment and must start less than 7 days following randomisation. Day of randomisation will be considered as day 1 for scheduling purposes. (3) Clinical assessment consists of complete history and examination at screening and focused history and relevant examination at subsequent visits. (4) Vital signs to include heart rate, blood pressure, temperature and weight. (5) Screening blood tests as detailed in section 5. (6) Standard blood tests consists of full blood count, urea and electrolytes, liver function tests, magnesium and alanine aminotransferase (ALT) international normalised ratio (INR). (7) Adverse effects and clinical events will be monitored continuously until completion of follow-up. Serious adverse events (SAE's) will be reported from the date of consent. All adverse events experienced by patients will be recorded irrespective of the causality (see section 7). (8) Mandatory microbiological testing must be performed between 7 and 30 days prior to leukapheresis—HBV, HCV, HIV, human T-lymphotrophic virus 1 and 2 (HTLV-1, HTLV-2) and syphilis. (9) The first re-infusion of CD133+ (group 3 patients only) will occur on one occasion only between days 6 and 10. The timing will be determined by the timing of each patient’s leukopheresis. CD133+ cell isolation and the local hospital arrangements (see section 7.6 in the study protocol).

The primary outcome measure will be change in δ MELD score at 90 days from baseline (figure 2). The δ MELD will be the difference between the MELD score at baseline and day 90 with a positive δ MELD indicating a worsening of the liver disease and a negative δ MELD an improvement.

MELD was initially developed to determine risk of mortality within 3 months in patients with cirrhosis undergoing transjugular intrahepatic portosystemic shunt (TIPS) insertion,^25^ but has subsequently been validated in outpatients with compensated cirrhosis^26^ and across a broad spectrum of liver disease.^27^ The MELD score is accurate in predicting 1 week, 3-month and 1 year mortality and is an independent predictor of clinical decompensation in patients with compensated cirrhosis.^28^ MELD score is used by all the major Western regulatory authorities involved in liver transplantation (UK Transplant, Eurotransplant and UNOS) to help prioritise the allocation of liver transplants.

Recent studies have shown that the change in MELD score over time to be a better predictor of outcome than the absolute or initial MELD score.^29–31^ In particular it has been shown that at any MELD score the magnitude and direction of change in MELD over the previous 30 days is a significant independent predictor of mortality.^30^ An increasing MELD over 3 months is also associated with the onset of hepatic decompensation in the form of variceal bleeding, ascites and encephalopathy.^31^ ^32^

The MELD score is thus a well-established prognostic scoring system for assessing the severity of chronic liver disease. MELD score is calculated using objective variables that are readily obtained namely serum bilirubin, serum creatinine and international normalised ratio (INR). These results will be used to calculate MELD scores using the accepted UNOS calculation, corrected for UK units of measurement:^26^
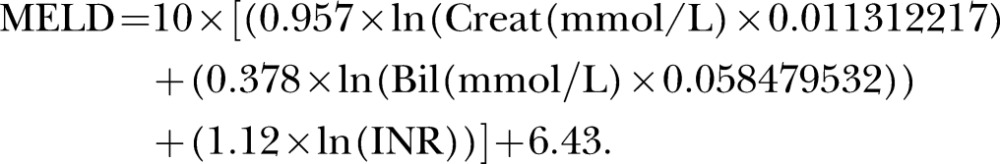


The following standard caveats will apply:

- Any value less than one will be given a value of 1.0 once converted to US units, to avoid a negative MELD score.
- If the patient has been dialysed twice in the past 7 days then the value for creatinine will be 4.0.
- The maximum MELD score is 40; all values greater than 40 are given a score of 40. (http://optn.transplant.hrsa.gov/converge/resources/MeldPeldCalculator).

## Secondary outcome measures

The time points at which the following secondary outcome measures will be recorded are shown in figure 2.

### Liver stiffness evaluation

Liver stiffness evaluation will be assessed by transient elastography (Fibroscan, Echosens, France), a non-invasive method for assessing liver fibrosis. Mild amplitude and low frequency vibrations (50 Hz) are transmitted to the liver tissue, inducing an elastic shear wave that propagates through the underlying liver tissue. The velocity of the wave is directly related to tissue stiffness, considered as an index of the amount of fibrotic tissue. This is expressed as a numerical value in kilopascals (kPa). The accuracy and reproducibility of transient elastography has been validated in chronic liver disease.^33^

### Enhanced liver fibrosis test

This is a validated panel of highly sensitive ELISA assays measuring matrix components and enzymes involved in their turnover: hyaluronic acid, tissue inhibitor of matrix metalloproteinase 1 and procollagen type III. The values for each of these markers is combined in an algorithm which produces a discriminant score (the ELF score) related to the level of liver fibrosis. The enhanced liver fibrosis (ELF) test (iQur, Southampton, UK) is accurate in assessing liver fibrosis in a range of chronic liver diseases and is a sensitive, specific and reproducible method for the non-invasive assessment of hepatic fibrosis.^34^

### Chronic liver disease questionnaire

This is a liver-specific questionnaire for measuring health-related quality of life in patients with chronic liver disease. It includes 29 items divided into 6 quality of life domains: abdominal symptoms, fatigue, systemic symptoms, activity, emotional function and worry. These items are ranked on a 1–7 scale, providing a possible range of scores from 29 (worst quality of life) to 203 (best quality of life). Validity has been demonstrated in all forms of chronic liver disease and test–retest reliability has been shown to be good.^35^ The chronic liver disease questionnaire (CLDQ) is self-administered and is designed to reflect the 2 weeks prior to testing.

### Individual components of liver function

This will include bilirubin, albumin, INR and creatinine values.

### UK end-stage liver disease score

The UK end-stage Liver Disease (UKELD) score is a scoring system developed by the UK Liver Transplant Units to predict transplant waiting list mortality.^36^ The score uses the parameters of bilirubin (Bil), INR, creatinine (Creat) and sodium (Na) as follows:
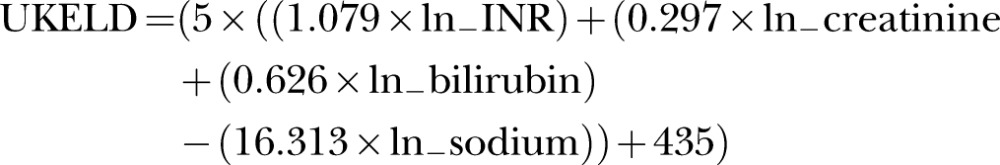


INR, serum creatinine and bilirubin values less than 1 are capped at 1.

Serum creatinine values greater than 400 are capped at 400.

Sodium values outside the range 112–150 are capped at the lower (112) and upper (150) limits.

### Circulating peripheral blood HSC

There are limited data on the effectiveness of GCSF-induced stem cell mobilisation in patients with cirrhosis, so this trial will enable a more detailed assessment of the safety and efficacy of achieving this. The numbers of circulating CD133+ HSC and circulating CD34+ HSC in peripheral blood will be quantified by flow cytometry according to ISHAGE protocols.^37^

### Clinical events and transplant-free survival

Liver-related clinical events will be recorded during the 12-month period of trial participation according to the following criteria: ascites (new development of clinically significant ascites or the worsening of established ascites), encephalopathy (requiring introduction of treatment or hospitalisation), portal hypertensive bleeding (confirmed at endoscopic examination), spontaneous bacterial peritonitis (PMN cell count >250 cm^3^ in ascitic fluid), development of hepatorenal syndrome, listing for liver transplantation, liver transplantation, development of hepatocellular carcinoma or dysplastic hepatic nodules and death. Transplant-free survival is defined as the interval between the date of randomisation and the date of transplant or death from any cause. Surviving transplant-free patients will be censored at date last seen alive.

## Statistical analysis

### Sample size justification

The primary outcome measure is the change in MELD score from baseline to 90 days postrandomisation. Analysis of 60 patients eligible for the trial in our clinic cohort demonstrated a mean baseline MELD score of 13.5, with a mean change in MELD over 3 months of 0.0±1.0. This indicates that no change in MELD over 90 days would be observed in conventionally treated patients. A clinically significant reduction would be at least a 1 point reduction in MELD. The trial is designed as a three-armed study with one control arm. The trial is powered to answer two hypotheses of each treatment compared to control but not powered to detect smaller expected differences between the two treatment groups. As such, the overall α=0.1 for the trial is split equally between the two hypotheses. Conventionally, to detect a relevant standardised effect size of 0.8 point reduction in MELD score using two-sided α=0.05 (overall α=0.1 split equally between the two hypotheses) and 80% power requires 27 participants to be randomised per group (81 participants in total; 49). The null hypothesis to be tested is that the change in mean at 90 days compared across two groups (treatment vs control) is the same. The alternative hypothesis is that the change in mean at 90 days compared across two groups (treatment vs control) is at least 0.8 SDs apart.

The number of participants lost to follow-up, or withdrawn consent prior to initial treatment is expected to be minimal. The Data Monitoring Committee (DMC) may advise replacement of participants if numbers are higher than anticipated.

## Analysis of primary outcome measure

All analyses will be carried out on an intention-to-treat basis, retaining participants in their randomised treatment groups and including protocol violator and ineligible participants, in order to maintain the unbiased comparison of treatments created by the randomisation procedure.

Baseline MELD score will be collected at randomisation and days 30, 60 and 90 postrandomisation. Change in MELD will be calculated for each participant and average changes presented and statistically compared for each treatment group against control using 2-sample t test or non-parametric two-sample Wilcoxon test if appropriate.

Changes from baseline for secondary outcomes scored as continuous measures will be calculated and presented descriptively by treatment group. It is unknown over what period the treatment remains active or if its effect increases linearly or in some other way. As such the statistical analysis plan will be updated to include an additional coprimary final analysis entailing a repeated measures analysis over time, specifically a mixed effect modelling procedure, taking into account the MELD measurement captured at baseline, days 30, 60 and 90. The introduction of mixed effects modelling will make more efficient use of the patient level information, enhance the scope of the study to understand the trend of treatment activity in more detail (rather than focusing only at a specific time point, day 90) and address missing values. To model the trend in MELD over time, a linear mixed effects models (taking into account within subject correlation) using linear, quadratic polynomials or more flexible semiparametric models will be considered. Goodness of fit tests will be used to compare the different models. We will evaluate if MELD changes over time, and if so, what is the pattern of change, as well as if the pattern differs between the each treatment and control group. For this analysis the null hypothesis to be tested is that the slope of the change in MELD compared across two groups (treatment vs control) is the same. The alternative hypothesis is that the slope of the change in MELD compared across two groups differ.

The multilevel coprimary analyses detailed in the foregoing await approval from the MHRA, as the REALISTIC trials team have sought consent to amend the primary analyses in this manner and believe the proposed changes will not be contested. The original powered analysis, the two sample t test, remains part of the primary analyses.

## Discussion

There is a clear need for new therapies for chronic liver disease as liver transplantation is limited by donor organ availability and by long-term complications. It has been proposed that BM-derived stem cell therapy may provide a therapeutic option and evidence from preclinical models has been encouraging.^7^ ^8^ The small scale studies performed in humans to date suggest the possibility of a beneficial effect in patients with liver disease but this has not been robustly tested in randomised controlled trials.^3^ The REALISTIC trial was designed to examine the benefits of repeated injections of bone marrow derived CD133+ stem cells in patients with cirrhosis and is the first randomised controlled trial with a powered primary end point to examine the safety and efficacy of repeated cell injections.

In the design of the trial a variety of experimental approaches were considered, in particular the route by which cell therapy would be administered and the primary outcome measure by which the efficacy of the cell therapy would be determined.

Administration of cells directly into the vessels supplying the liver (portal vein and hepatic artery) is invasive and carries significant risks, including bleeding and thrombosis. This approach had been used in initial studies without complication, however, subsequent studies have shown this to be complicated by portal hypertensive bleeding following cell injection^38^ and one study was terminated early following complications in two out of four patients after hepatic artery injection.^39^ In this trial HSC will be administered into a peripheral vein thus avoiding the potential complications of repeated procedures to access hepatic vessels. In addition to reducing the risks of cell injection, this approach has practical implications in making the therapy more accessible and cost-effective as specialised interventional radiological procedures are not required.

In patients with compensated cirrhosis expected mortality at 1 year is between 1% and 3% with median survival of 12 years, with complications of cirrhosis developing at a rate of 5–7% per year.^40^ The low short-term mortality in these patients means that utilising survival as the primary outcome measure would not be appropriate. MELD score is an objective, prognostic scoring system validated for patients with chronic liver disease, and the variables required to calculate it are easily obtained from standard blood tests. The magnitude of change in MELD score is predictive of the development of the complications of cirrhosis, and in this trial the change in MELD score at 90 days will be used as the primary outcome measure. The authors hope such a trial design will fill an important gap in the current literature of cell therapy for liver disease. Furthermore such a design may be a template for future studies with other novel cell therapy products which are undergoing development for the therapy of chronic liver disease.
